# Epidemiological Aspects of Notified Sexually Transmitted Infections and a Review of the Current Surveillance System in Tabuk, Saudi Arabia

**DOI:** 10.7759/cureus.76707

**Published:** 2024-12-31

**Authors:** Hamza M Omer, Ghormallah A Al Ghamdi, Mubarak M Alsaeed, Faris F Alanazi, Mohammad Al Mamun

**Affiliations:** 1 Department of Public Health, Ministry of Health, Tabuk, SAU

**Keywords:** epidemiology, notification, saudi arabia, sexually transmitted infections, surveillance

## Abstract

Objective

Our study aimed to identify the epidemiological patterns of notified sexually transmitted infections (STIs) in the Tabuk Region, Saudi Arabia, and to review the existing STI surveillance system.

Methods

We conducted a retrospective cross-sectional study, extracting STI surveillance data from the Health Electronic Surveillance Network Plus (HESN Plus) database. All notified STI cases (100%) reported to the Department of Public Health, Ministry of Health in the Tabuk Region, and available in the HESN Plus database until 19 November 2024, were included in the study. The extracted data included information on the types of STIs, diagnoses, and various demographic and epidemiologic details of the patients. Data analysis was performed using SPSS 16.0 (SPSS, Chicago, IL) for Windows, employing the Chi-square test to explore associations between categorical variables. The current STI surveillance system was reviewed using specific criteria, including data completeness and the status of case investigations.

Results

Of the total 290 STI cases recorded in the HESN Plus system, etiologic STIs accounted for a higher proportion (210, 72.4%) compared to syndromic STIs (80, 27.6%). Vulvovaginal candidiasis was the most common etiologic STI, comprising 102 (35.2%) of the total cases, while urethral discharge syndrome was the most prevalent syndromic STI, representing 43 (14.8%) of the total. Overall, STIs were predominant among females (188, 64.8%), Saudi citizens (245, 88.4%), and middle-aged adults (138, 47.6%). The mean age of notified STI cases was 33.69±10.9 years. The majority of STIs were notified by hospitals (154, 53.1%), with the remaining cases reported by various health centers (136, 46.9%). Significant associations were found in the distribution of etiologic and syndromic STI cases by gender (p<0.001) and type of health facility (p=0.026). A remarkable proportion of records had missing data on epidemiological links (287, 99.0%) and signs and symptoms (283, 97.6%). Furthermore, 131 (45.2%) of cases were still open, indicating incomplete epidemiological or clinical investigations.

Conclusion

This study highlights the considerable burden of STIs in the Tabuk Region, with a higher proportion of etiologic STIs compared to syndromic STIs. The current STI surveillance system reveals substantial gaps in data completeness, especially concerning epidemiological links and signs and symptoms. These deficiencies underscore the need for improved STI reporting practices and suggest updating the data entry process by incorporating mandatory data fields within the HESN Plus system to ensure the quality of STI surveillance data. It is crucial to ensure comprehensive training for healthcare providers, including surveillance officials, and to implement targeted STI-related interventions focusing on high-risk demographics.

## Introduction

Globally, sexually transmitted infections (STIs) remain a significant public health challenge [1]. According to the World Health Organization (WHO), more than one million curable STIs are acquired every day among people aged 15-49, with the majority being asymptomatic [2]. In 2022, there were an estimated eight million adults in this age group infected with syphilis [2], and over 500 million people aged 15-49 were estimated to have genital infections with herpes simplex virus (HSV) [3]. Efforts to reduce new infections and improve treatment and prevention strategies are ongoing, but challenges remain [1,4].

Similar to the global trend, STIs are also a substantial public health concern in Saudi Arabia. Recent studies have documented a rising prevalence of STIs, attributed to increased urbanization and evolving sexual behaviors [5-7]. The prevalence of STIs, including nongonococcal urethritis, trichomoniasis, syphilis, genital warts, and gonorrhea, has notably increased [5]. This upward trend underscores the urgent need for robust surveillance and effective public health interventions.

Recent studies have shed light on various aspects of STI awareness and barriers in Saudi Arabia. Alomair et al. conducted qualitative research exploring Muslim women's knowledge, views, and attitudes toward STIs, highlighting a significant gap in awareness [6]. Furthermore, Alomair et al. identified key barriers to STI testing and diagnosis from Muslim women's perspectives, emphasizing cultural and societal obstacles [8]. Additionally, Alotaibi et al. examined the association of STIs and human papillomavirus (HPV) co-infection with abnormal cervical cytology among women, contributing to the understanding of STI-related complications [7]. Comprehensive studies by Al-Sahli et al. and Kabbash et al. also underscore the need for improved awareness and management of STIs in Saudi Arabia [9,10].

STI surveillance systems are crucial for monitoring and controlling the spread of STIs globally [2]. These systems collect, analyze, and disseminate data on STI cases, helping public health officials identify trends, allocate resources, and implement effective prevention and control measures [11]. Effective STI surveillance systems, such as those recommended by CDC, UNAIDS, and WHO, are essential for guiding public health initiatives and reducing the burden of STIs [11,12].

In an earlier study, STI case notification rates in Saudi Arabia highlighted the critical importance of continuous surveillance and public health initiatives [5]. However, the current STI surveillance system's effectiveness and limitations remain underexplored, particularly in the Tabuk Region. By identifying the system's shortcomings, this research provides valuable insights for improving STI surveillance strategies and public health interventions, ultimately aiming to reduce the STI burden in the region. The primary objective of this study was to identify the epidemiological aspects of notified STIs in the Tabuk Region and to review the existing STI surveillance system. The secondary objectives included analyzing demographics to examine the distribution of STIs across different age groups, genders, and nationalities; determining the presence of associations between STI case types and patients' background characteristics; and assessing the reporting practices for consistency and completeness in the data reported within the electronic STI surveillance system.

## Materials and methods

Study design

This study employed a retrospective cross-sectional design. This study design involved analyzing data that were collected at a single point in time from past records.

Data collection

The research team accessed the Health Electronic Surveillance Network Plus (HESN Plus) database and extracted STI surveillance data. This database, maintained by the Department of Public Health, Ministry of Health in the Tabuk Region, contains detailed information on reported STI cases. These electronic records provided a comprehensive source of reported STI cases. The extracted data included information on the types of STIs, diagnoses, and various demographic and epidemiologic details of the patients.

Data extraction timeframe

The data were extracted from the HESN Plus, covering the period from 7 November 2022 to 19 November 2024.

Sampling method and sample size

All notified STI cases (100%) reported to the Department of Public Health, Ministry of Health in the Tabuk Region, and available in the HESN Plus database until 19 November 2024, were included in the study.

Data analysis

Data analysis was performed using SPSS 16.0 for Windows (SPSS, Chicago, IL). Descriptive analysis was conducted to determine the frequency distribution and percentage of various types of STIs. Chi-square tests were conducted to determine the presence of associations between STI case types (etiological and syndromic) and various background characteristics. These characteristics include a) gender: male and female, b) nationality: Saudi and non-Saudi, c) age category: various age groups, and d) types of health facilities: hospitals and health centers. Results were considered statistically significant if the p-value was less than 0.05. This analysis identified demographic patterns among the patients and determined potential factors associated with STIs. The results were presented in the form of tables to highlight the findings.

Review of the surveillance system

The current STI surveillance system was reviewed using specific criteria, including data completeness and the status of case investigations. This review aimed to assess the existing HESN Plus system, providing a basis for recommending improvements.

Ethical considerations

Data were extracted anonymously from the HESN Plus system, ensuring that names, national ID numbers, and phone numbers of patients were not collected. All data were handled with strict confidentiality to protect the privacy and anonymity of patients. Ethical approval for this study was obtained from the Tabuk Institutional Review Board, Ministry of Health, Tabuk Region, Kingdom of Saudi Arabia (TU-077/024/267; Date: 31 October 2024). Informed consent from the patients was waived because this study was a record-based retrospective study, in which the data were extracted anonymously without identifying personal or private information.

## Results

**Figure 1:**
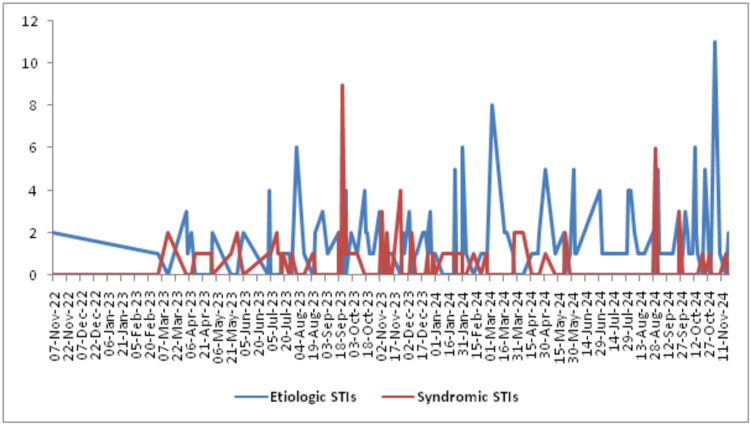
Epidemiological curve of notified STIs (n=290) by etiologic and syndromic case types in Tabuk, Saudi Arabia STIs, sexually transmitted infections

The HESN Plus system showed that STI surveillance data has been included in its database for the Tabuk Region since 7 November 2022. As of 19 November 2024, a total of 290 STI cases were recorded in the HESN Plus system, all of which were included in the analysis. Figure 1 illustrates the epidemiological curve of notified STIs by etiologic and syndromic case types in Tabuk, Saudi Arabia. It indicates that the occurrence of etiologic STIs was higher than that of syndromic STIs.

Table 1 shows the diagnosis pattern of notified STIs in Tabuk, Saudi Arabia. Out of the 290 STI cases, the majority (210, 72.4%) were etiologic in nature, while the remaining (80 (27.6%)) were reported as syndromic. The most frequently diagnosed etiologic STI was vulvovaginal candidiasis, comprising 102 (35.2%) of the total cases. Other notable etiologic STIs included syphilis (32, 11%), gonorrhea (22, 7.6%), and genital warts (15, 5.1%). Among syndromic STIs, urethral discharge syndrome was the most prevalent, accounting for 43 (14.8%) of the total cases. The second most prevalent syndromic STI was vaginal discharge syndrome (27, 9.3%).

**Table 1:** Diagnostic pattern of notified STIs (n=290) in Tabuk, Saudi Arabia n, total number of STI cases; %, percentage of n; STIs, sexually transmitted infections

Type of STI Cases	Diagnosis	Number (%)
Etiologic	Vulvovaginal candidiasis	102 (35.2)
	Syphilis	32 (11.0)
	Gonorrhea	22 (7.6)
	Genital warts (human papillomavirus)	15 (5.1)
	Bacterial vaginosis	14 (4.8)
	Trichomoniasis	14 (4.8)
	Pelvic inflammatory disease	4 (1.4)
	Nongonococcal urethritis	3 (1)
	Chlamydial genital infections	2 (0.7)
	Genital herpes simplex virus	2 (0.7)
Total		210 (72.4)
Syndromic	Urethral discharge syndrome	43 (14.8)
	Vaginal discharge syndrome	27 (9.3)
	Genital ulcer syndrome	4 (1.4)
	Lower abdominal pain syndrome	4 (1.4)
	Genital warts syndrome	2 (0.7)
Total		80 (27.6)

Table 2 presents the background characteristics of notified STIs by etiologic and syndromic case type in Tabuk, Saudi Arabia. Overall, the majority of STI cases were female (188, 64.8%) and Saudi citizens (245, 88.4%). The most prevalent age group was 31-50 years, accounting for 138 (47.6%) of the cases, followed closely by those aged 18-30 years (120, 41.4%). The mean age was 33.69 years, with a standard deviation of 10.9 years, and the ages ranged from one to 80 years. The majority of STIs were notified from hospitals (154, 53.1%), while the remaining cases were reported by various health centers (136, 46.9%).

**Table 2:** Background characteristics of notified STIs by etiologic and syndromic case types in Tabuk, Saudi Arabia n, total number of STI cases; %, percentage of n; STIs, sexually transmitted infections; PHCCS, primary health care centers *Data not available for 13 case **Includes Sudanese, Pakistani, Yemeni, Egyptian, Indian, Nepali, Jordanian, and Syrian ***Government and private ****Includes government PHCCS, private clinics, private diagnostic laboratories, and the Tabuk Prison Health Center

Characteristics	Category	STI Cases			Significance (χ^2^ Test P-Value)
		Total (%)	Etiologic (%)	Syndromic (%)	
Gender (n=290)	Male	102 (35.2)	55 (19.0)	47 (16.2)	<0.001
	Female	188 (64.8)	155 (53.4)	33 (11.4)	
Nationality (n=277)*	Saudi (citizen)	245 (88.4)	179 (64.6)	66 (23.8)	0.212
	Non-Saudi (resident)**	32 (11.6)	20 (7.2)	12 (4.3)	
Age in years (n=290)	<18	11 (3.8)	9 (3.1)	2 (0.7)	0.877
	18-30	120 (41.4)	88 (30.3)	32 (11.0)	
	31-50	138 (47.6)	98 (33.8)	40 (13.8)	
	>50	21 (7.2)	15 (5.2)	6 (2.1)	
Type of health facility reported cases (n=290)	Hospital***	154 (53.1)	120 (41.4)	34 (11.7)	0.026
	Health Center****	136 (46.9)	90 (31.0)	46 (15.9)	

Table 2 also illustrates that females were more frequently diagnosed with etiologic STIs (155, 53.4%) compared to males (55, 19%). Similarly, Saudi citizens represented a higher proportion of both etiologic (179, 64.6%) and syndromic (66, 23.8%) STIs compared to non-Saudis. The age group 31-50 years showed the highest proportion of both etiologic (98, 33.8%) and syndromic (40, 13.8%) STIs. Regarding health facilities, hospitals reported a higher number of etiologic cases (120, 41.4%), while health centers reported a larger number of syndromic cases (46, 15.9%).

There were significant differences in the distribution of etiologic and syndromic STI cases by gender (p<0.001). Similarly, significant associations were found between the type of STIs and the type of health facility reported in the cases (p=0.026). However, no significant differences were observed between Saudi citizens and non-Saudis in the distribution of etiologic and syndromic cases (p=0.212). Furthermore, no significant associations were found among the age groups in the distribution of etiologic and syndromic cases (p=0.877).

Table 3 presents the status of data incompleteness of notified STI cases, highlighting considerable gaps in data entry and case investigation status in Tabuk, Saudi Arabia. In the HESN Plus dataset, 13 (4.5%) of the records lacked information on the nationality of the cases. Additionally, 33 (11.4%) of the records did not specify whether the individuals were cases or contacts (sex partners). A remarkable proportion of records had missing data on epidemiological links (287, 99%) and signs and symptoms (283, 97.6%). Furthermore, 131 (45.2%) of cases were still open, reflecting incomplete epidemiological or clinical investigations.

**Table 3:** Data incompleteness of notified STI cases (n=290) in the HESN Plus in Tabuk, Saudi Arabia n, total number of STI cases; %, percentage of n; HESN Plus, Health Electronic Surveillance Network Plus; STIs, sexually transmitted infections *Indicates incompleteness of data entry **Indicates incompleteness of epidemiological or clinical investigation of the case

Variable	Category	Number (%)
Nationality	Saudi	245 (84.5)
	Non-Saudi	32 (11.0)
	Data not available*	13 (4.5)
Case or contact (sex partner)	Case	254 (87.6)
	Contact (sex partner)	3 (1.0)
	Data not available*	33 (11.4)
Risk factor (epidemiological link)	Information available	3 (1.0)
	Information not available*	287 (99.0)
Signs and symptoms	Chancre	2 (0.7)
	Vaginal discharge	2 (0.7)
	Urethral discharge	2 (0.7)
	Ulcer on penis, scrotum, and rectum	1 (0.3)
	Information not available*	283 (97.6)
Case investigation status	Closed	159 (54.8)
	Open**	131 (45.2)

## Discussion

The present study identified vulvovaginal candidiasis as the most common etiologic STI (102, 35.2%) and urethral discharge syndrome as the most frequent syndromic infection (43, 14.8%). The higher occurrence of vulvovaginal candidiasis among women may be attributed to factors such as hygiene practices, awareness levels, healthcare-seeking behaviors, and the accessibility of healthcare facilities. Additionally, the increased frequency observed in our study suggests the need for further investigation into underlying causes, including specific risk factors like diabetes and obesity, especially within the context of Saudi Arabia, where these conditions are known to increase susceptibility to fungal infections. Memish et al. reported nongonococcal urethritis (35613, 51.7%) and trichomoniasis (12679, 18.4%) as the predominant etiologic STIs in Saudi Arabia [5], highlighting a divergence in the predominant infections between our study and theirs. Our finding of urethral discharge syndrome as the most common syndromic STI aligns with the general trend observed by Memish et al., although the specific etiologies differ. Moreover, while Kabbash et al. reported vaginal discharge syndrome (4,170 cases, 77.6%) as the most common syndromic infection in their study [10], our results showed a lower frequency of this syndrome compared to urethral discharge syndrome. This discrepancy might be due to regional variations and differences in population characteristics. Furthermore, a study within the African cohort reported a 7.7% (273 cases) prevalence of syndromic STIs, with higher rates among females and young individuals [13]. In contrast, our findings indicated a higher proportion of etiologic STIs in females and young age groups. This difference could be attributed to variations in diagnostic facilities, reporting practices, or sample sizes.

Our study showed a higher proportion of etiologic STIs in females compared to males. This aligns with global trends, where women are often more frequently diagnosed with STIs [14] due to factors like biological susceptibility [15], limited access to healthcare [16], and lack of awareness and education [16]. The age group 31-50 years showed the highest proportion of both etiologic and syndromic STIs in our study. This is similar to findings from other literature that have identified higher STI rates in middle-aged adults due to their sexual activity patterns and increased risk behaviors [17,18]. Hospitals reported a higher number of etiologic cases, while health centers reported a larger number of syndromic cases. This is typical because hospitals have better laboratory diagnostic facilities than health centers. This finding suggests that different types of health facilities may have varying capacities for diagnosing and reporting different types of STIs [19]. Our study found significant differences in the distribution of etiologic and syndromic STI cases by gender and health facility type. This indicates that various demographic factors and the types of health facilities can influence the diagnosis and reporting of STIs [20,21]. This finding also suggests the need for targeted interventions, particularly focusing on females and the types of health facilities providing care.

The HESN, a web-based surveillance system launched by the Ministry of Health in Saudi Arabia, was later upgraded to HESN Plus and activated across all health facilities in early 2022 [22]. This system aims to enhance public health surveillance by providing real-time, precise data on communicable diseases and epidemics [23]. It helps decision-makers and public health practitioners prevent disease outbreaks and improve public health outcomes by unifying health processes and minimizing disparities in surveillance data [23].

However, our study highlights substantial gaps in the data entry and case investigation processes for STIs within the HESN Plus system in the Tabuk Region. The HESN Plus dataset revealed that some records lacked information on the nationality of cases, while others did not specify whether individuals were cases or contacts (sex partners). Additionally, a majority of the records had missing data on risk factors or epidemiological links and lacked information on signs and symptoms. The main reason for these gaps is the lack of a mandatory data entry option for these specific variables in the HESN Plus system, allowing individuals to skip these fields and proceed to the next step without entering data. Furthermore, a considerable portion of cases remained "open," indicating that epidemiological or clinical investigations were incomplete. These findings underscore an urgent need for improved data collection, entry, and reporting practices to ensure comprehensive surveillance and effective public health interventions.

Our study has several limitations. Although the inclusion criteria for "notified STI cases" in the HESN Plus system were applied uniformly across all health facilities, the reliance on surveillance data poses challenges, such as potential underreporting or misclassification of STIs, which may bias the findings. Additionally, the study focused on the Tabuk Region, restricting the generalizability of the results, as the data may not be representative of the entire country of Saudi Arabia. We were unable to explore some socioeconomic, cultural, and behavioral factors that may have influenced the occurrence of STIs because the HESN Plus system did not include mandatory fields for recording these data. Furthermore, variations in the availability and quality of STI services in different healthcare facilities could impact the surveillance data.

## Conclusions

The present study found a higher proportion of etiologic STIs compared to syndromic STIs in the Tabuk Region. Overall, STIs were predominant among females and middle-aged adults. Among etiologic STIs, vulvovaginal candidiasis was the most frequent infection, while among syndromic STIs, urethral discharge syndrome was the most common. Hospitals reported a greater number of etiologic cases, whereas health centers reported more syndromic cases. Significant associations were found in the distribution of etiologic and syndromic STI cases by gender and type of health facility. The study also revealed substantial gaps in the data entry and case investigation processes within the HESN Plus system, emphasizing the urgent need for improved reporting practices and the inclusion of mandatory reporting fields or options. Ensuring the accuracy and completeness of surveillance data requires better reporting practices in all local health settings. Additionally, incorporating a robust monitoring system by the regional health authority is crucial. To enhance the effectiveness of STI surveillance and public health interventions, we recommend addressing existing data gaps, ensuring comprehensive training for healthcare providers, including surveillance officials, and implementing targeted STI-related interventions focusing on high-risk demographics, particularly women and middle-aged individuals. It is also essential to increase public awareness and address the stigma related to STIs through community-based interventions.
